# Effect of Needle Insertion Speed on Tissue Injury, Stress, and Backflow Distribution for Convection-Enhanced Delivery in the Rat Brain

**DOI:** 10.1371/journal.pone.0094919

**Published:** 2014-04-28

**Authors:** Fernando Casanova, Paul R. Carney, Malisa Sarntinoranont

**Affiliations:** 1 Department of Mechanical & Aerospace Engineering, University of Florida, Gainesville, Florida, United States of America; 2 Department of Pediatrics, Neurology, Neuroscience, and J. Crayton Pruitt Family Department of Biomedical Engineering, Wilder Center of Excellence for Epilepsy Research, Gainesville, Florida, United States of America; 3 Escuela de Ingeniería Mecánica, Universidad del Valle, Cali, Colombia; University of Arizona, United States of America

## Abstract

Flow back along a needle track (backflow) can be a problem during direct infusion, e.g. convection-enhanced delivery (CED), of drugs into soft tissues such as brain. In this study, the effect of needle insertion speed on local tissue injury and backflow was evaluated *in vivo* in the rat brain. Needles were introduced at three insertion speeds (0.2, 2, and 10 mm/s) followed by CED of Evans blue albumin (EBA) tracer. Holes left in tissue slices were used to reconstruct penetration damage. These measurements were also input into a hyperelastic model to estimate radial stress at the needle-tissue interface (pre-stress) before infusion. Fast insertion speeds were found to produce more tissue bleeding and disruption; average hole area at 10 mm/s was 1.87-fold the area at 0.2 mm/s. Hole measurements also differed at two fixation time points after needle retraction, 10 and 25 min, indicating that pre-stresses are influenced by time-dependent tissue swelling. Calculated pre-stresses were compressive (0 to 485 Pa) and varied along the length of the needle with smaller average values within white matter (116 Pa) than gray matter (301 Pa) regions. Average pre-stress at 0.2 mm/s (351.7 Pa) was calculated to be 1.46-fold the value at 10 mm/s. For CED backflow experiments (0.5, 1, and 2 µL/min), measured EBA backflow increased as much as 2.46-fold between 10 and 0.2 mm/s insertion speeds. Thus, insertion rate-dependent damage and changes in pre-stress were found to directly contribute to the extent of backflow, with slower insertion resulting in less damage and improved targeting.

## Introduction

Successful treatment of neurological disorders requires effective techniques to deliver drugs to the central nervous system (CNS). Systemic delivery can be problematic because of the blood brain barrier (BBB) which prevents passive passage of the majority of large molecules from the bloodstream into the extracellular space [1], [2]. Convection-enhanced delivery (CED) is a technique to deliver therapeutic macromolecules directly to the CNS and bypass the BBB [3]. In CED, a cannula (needle) is inserted directly into the parenchyma, and drug infusate is delivered at controlled flow rates into the extracellular space. With convection (advection) as the dominant transport mechanism, this technique can improve local delivery by providing larger distribution volumes than diffusion-dependent methods [3].

CED requires the surgical insertion of a needle into brain tissue, and this raises specific concerns about minimizing penetration injury and ensuring proper targeting. Even with stereotactic positioning, backflow (leakback) along the needle track continues to be a major source of non-specific targeting, especially at high flow rates [4]. With backflow, infusate flows back along the outer needle wall instead of penetrating into tissue at the needle tip. This backflow phenomenon is normally undesirable since large tissue distributions are not achieved, and drugs can reach regions of the brain where they are unintended, not effective, or toxic, resulting in unwarranted side effects [5], [6]. Indeed, improper targeting has been implicated as one of the most significant barriers to successful implementation of CED in previous clinical brain tumor trials [6], [7]. In these trials, high rates of ineffective delivery resulted in the majority of infusate leaking into CSF spaces [5], [8]. These studies employed simple open-ended cannulas that are prone to backflow [8], [9]. To reduce backflow, research has focused on developing new cannula designs [10]–[12], imaging to identify backflow in real-time [13]–[18], and on modeling to predict the extent of backflow [19]–[21].

Backflow can be produced by local tissue injury, fluid-tissue interactions, or by a combination of these effects. With needle insertion, tissue disruption and injury may create a fluid-filled gap between the needle and surrounding tissue through which infusate can easily flow with little fluid resistance. Edema may also develop in response to cellular injury or vascular damage, and this abnormal accumulation of fluid can result in swelling and volumetric tissue changes [22]. Expansion of extracellular space may introduce a high hydraulic conductivity (permeability) layer of traumatized tissue around the cannula through which infusate can easily flow [23]. Alternatively, cellular swelling may reduce extracellular space and help to close gaps at the needle-tissue interface. Even when no fluid-filled gap is present at the needle interface, fluid pressure at the needle tip is increased with an increasing infusion flow rate, and this elevated pressure can push soft tissue away from the cannula surface to create a fluid-filled gap (intrinsic backflow) [20], [21]. Related to this mechanism, tissue coring, defined as tissue going inside the cannula during the insertion process, has being implicated as another cause of increased backflow [3], [14], [24]. Peaks in needle fluid pressure which may be required to clear the obstruction also result in a peak in fluid pressure at the needle tip potentially creating a bolus infusion and backflow.

Tissue stresses which are introduced or are pre-existing may also influence the mechanics of backflow. We define pre-stress as the stress that exists around the needle after insertion and before the start of CED infusion. We assume this stress is radially-directed along the needle-tissue interface, and it is created by tissue disruption, displacement, and subsequent compaction around the needle with insertion. The introduction of this stress may reduce backflow since fluid pressure would have to overcome it to separate the tissue from the needle surface [21]. In our previous studies, we have evaluated the effect of pre-stress on CED backflow in hydrogel tissue phantoms [25]. The presence of pre-stress resulted in reduced backflow. However, experimental measures or mechanical analysis of this stress is currently missing for CNS tissues. In CNS tissue, local tissue swelling around the needle can affect backflow if it produces changes in stress distribution before or during infusions. Therefore, local tissue swelling in response to needle injury can result in time-dependent pre-stress and backflow outcomes. In addition, it should be noted that residual stresses already exist in brain tissues even before needle insertion [26], [27]. Such residual stresses may also influence backflow by altering the mechanics of tissue disruption, changing fluid-gap dimensions, or changing the magnitude of pre-stress.

Studies of local CNS tissue penetration injury have been previously conducted for insertion of electrodes and needles and for ballistic tests. Blood vessel rupture and hemorrhage have been previously reported as evidence of tissue damage during electrode insertion in cortical tissues [28], [29]. Previous CED studies by White et al. [23] observed regions of increased tissue injury with hemorrhage reported mainly within white matter regions, and pressure-induced tissue fracture near the needle tip. Regions of injury were also found to be coincident with backflow. While there are no needle insertion studies measuring acute tissue swelling, early tissue swelling has been detected due to other kinds of brain trauma [30]–[32].

Few studies have focused on evaluating insertion speed on CNS tissue damage. Previous electrode studies have been conducted in which fast insertions of sharp electrodes and electrode arrays into cortical tissue were shown to cause less vascular rupture and bleeding [28], [29]. In our previous study, we have evaluated the influence of needle insertion speed on backflow in a hydrogel tissue phantom [25]. Backflow was considerably reduced if the needle was inserted fast (1.8 mm/s). A pre-stress was estimated, and the existence of this compressive interfacial stress was postulated to contribute to reduced backflow. At a lower speed (0.2 mm/s), hydrogel accumulation at the needle tip produced a damage gap that resulted in backflow. Thus, rate-dependent damage had a great influence on the extent of backflow.

In the CNS, we assume that surgical placement of the infusion cannula creates tissue damage and a volumetric loss of tissue. For example, as cell membranes are irreparably torn by needle penetration, the fluid content of each cell will disperse or be squeezed out into the interstitial space through which it can flow easily to other tissue regions. Also, large strains introduced by needle insertion may result in deformation that results in a permanent track observed along the needle pathway. In this study, the effect of the needle insertion speed on such tissue damage and CED backflow was evaluated *in vivo* in rat brain tissue. The caudate putamen, a gray matter region, was targeted with the needle track passing through both the cortex and external capsule, a white matter structure, before reaching target tissue. Local tissue damage due to needle coring, needle disruption/displacement, and time-dependent swelling was evaluated. The extent of tissue coring was evaluated by monitoring CED infusion pressures. Local tissue damage and time-dependent swelling in the vicinity of the needle were evaluated by histological hole measurements at two time points after needle retraction. Hole measurements were used to reconstruct the tissue penetration damage along the length of the needle track. Hole diameters were also used as an input into a mechanics model to estimate *in vivo* pre-stresses assuming brain tissue to behave as a neo-Hookean material. Finally, these damage assessments were used to understand the influence of rate-dependent injury and pre-stress on backflow. CED experiments infused visible Evans blue albumin (EBA) tracer at varying flow rates and insertion speeds. Backflow was quantified in two ways: percentage of tracer infusate outside the targeted caudate putamen and distance back along the needle track. This study provides a visual way to evaluate mechanical damage along the needle track. Also, to the best of our knowledge it is the first to quantify pre-stress in CNS tissues and study its relation with insertion rate and backflow. The results of this study may be used to better understand and improve the insertion processes of needles, electrodes, probes or other devices in the brain to reduce tissue injury and improve functional outcome.

## Methodology

Three series of *in vivo* rat brain experiments were conducted: (1) needle insertion experiments to measure local tissue damage along the needle track (infusion pressure measurements and hole measurements), *n* = 12 rats, (2) additional histological assessment, *n* = 3 rats, and (3) CED backflow experiments, *n* = 23 rats.

### Animal preparation and surgical procedures

All experiments were performed on male Sprague-Dawley rats (300–350 g) using protocols and procedures approved by the University of Florida Institutional Animal Care and Use Committee. Anesthesia was initiated with xylazine (10 mg/kg, SQ) and isoflurane (4%) in oxygen delivered at 1.0 L/min. The head was shaven and disinfected with iodine/alcohol. Then animals were placed in a stereotaxic frame (model 900, David Kopf Instruments, Tujunga, CA), and inhalation anesthesia (1.5% in 0.5 L/min of oxygen) was delivered via a nose mask. Body temperature was maintained (∼37°C) with a heating pad during the entire procedure. The skull was exposed by a mid-sagittal incision that began between the eyes and extended caudally to the level of the ears to expose the bregma and lambda. Two holes of 2 mm diameter were drilled by hand into the skull above the caudate putamen (CPu). Dura mater was carefully taken off and any residual blood was cleaned with phosphate-buffered saline (1× PBS). The exposed surface of the brain was kept wet with PBS during the course of the experiment.

**Figure 1:**
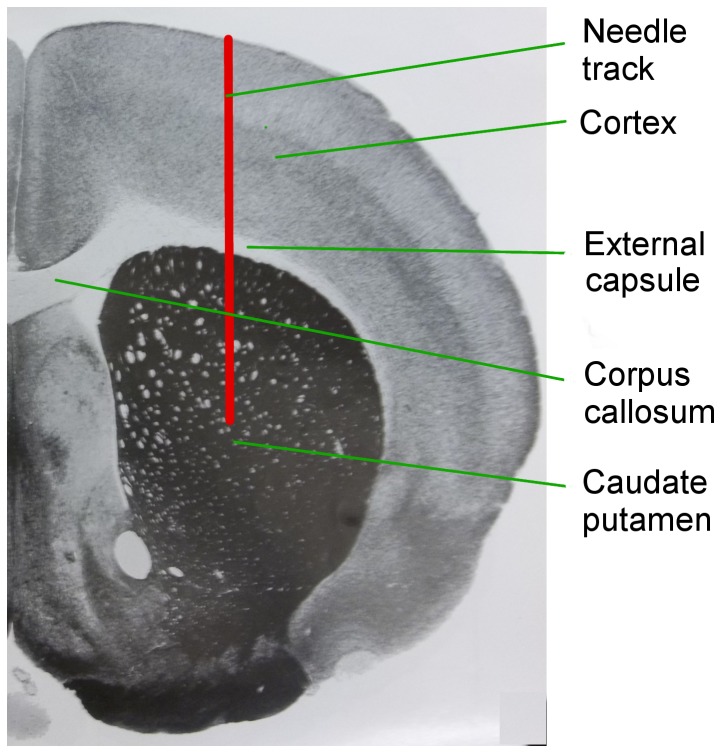
Coronal section of the rat brain schematically showing the needle track (in red) passing through the cortex and the external capsule and targeting the caudate putamen (CPu). Slice picture adapted from [41].

The CPu was chosen as the target because it is a relatively homogeneous region composed mainly of gray matter and has been previously used for CED backflow studies [4], [33]–[35]. To reach the CPu, the needle must pass through the cortex, which is mainly gray matter and the external capsule (ec) which is a white matter region (Figure 1). The needle was stereotactically inserted at a 5 mm depth within the CPu. Bilateral infusions were conducted on the right and the left sides (AP = 0.5, ML = ±3, DV = −5). After needle insertion, two minutes were allowed for tissue relaxation before infusions. The needle was retracted three minutes after the end of all infusions. The needle was retracted at 0.2 mm/s, and cleaned with 3% hydrogen peroxide followed by 1×PBS.

In needle insertion experiments and histology experiments, brain tissues were fixed to avoid further swelling after rat death and to avoid additional distortion and damage during removal and slicing. After needle retraction, rats were immediately euthanized by perfusion fixation using 10% formalin. Brains were removed and put in 10% formalin overnight and then moved to 30% sucrose for 2 days before tissue slicing. To be consistent with previous backflow studies [4], brains used for CED backflow experiments were not fixed, and rats were euthanized by decapitation immediately after needle retraction.

### Needle insertion and CED system

For all experiments, a 32 gauge (0.235 mm diameter) blunt tip stainless steel needle (Hamilton, Reno, NV) was used. Blunt tip needles were used because needles of this kind have been used in clinical trials [6], are readily commercially available, and have been used by our lab in previous CED studies [13], [14], [25]. Moreover, even though new cannulas to minimize backflow have being designed [10]–[12], the tip geometry of many of these new designs is often a basic blunt tip.

**Figure 2:**
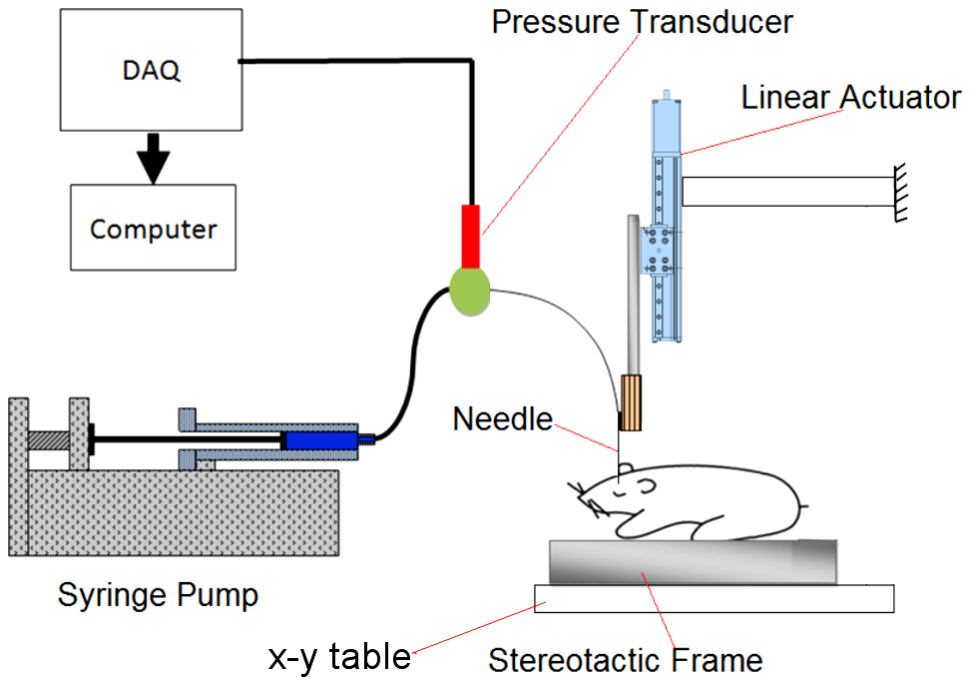
Schematic of the experimental set up used for needle insertion and CED.

Figure 2 shows a schematic of the experimental set-up used for needle insertion and CED. Needle insertion (*z*-direction) was controlled using a linear stage system (model LP28T, Applied Motion Products, Watsonville, CA) mounted on a machine frame. The needle was coupled to the linear stage using an electrode holder (model 900, David Kopf Instruments, Tujunga, CA) which also provided alignment. The stereotaxic frame and rat were placed on a *x-y* table which was used to position the rat under the needle such that the drilled holes in the rat skull at target AP and ML coordinates was under the needle tip. The infusion system consisted of a syringe pump driving a 100 µL gas-tight syringe (Hamilton, Reno, NV) connected to 40 cm of minimally compliant polyetheretherketone (PEEK) tubing (1 mm inner diameter and 1.58 mm outer diameter). This infusion line was coupled to the needle via a reducing union (Valco Instruments, Houston, TX).

### Macromolecular tracer

Evans blue labeled albumin (EBA) was used as the visible macromolecular tracer. EBA was prepared with 20 mg/ml of bovine serum albumin (Sigma-Aldrich, St. Louis, MO) and 1.25 mg/ml of Evans blue (Sigma-Aldrich, St. Louis, MO) in 1× PBS. The concentration of Evans blue was low enough that Evans blue was completely bound to the albumin [37].

### Needle insertion experiment

Experiments were conducted at three insertion speeds: 0.2, 2, and 10 mm/s. Four rats were tested at each insertion speed (12 total rats). The first two speed levels were chosen because they are in the range of a previous electrode insertion study [28] and similar to the speed levels of our previous hydrogel CED study [25]. The highest insertion speed was chosen based on a previous microelectrode study [29]. 4 µL of tracer was infused at 2 µL/min to reproduce conditions of backflow and to provide easier identification for hole measurements in fixed tissue sections.

To evaluate changes in our damage parameter, hole diameter, due to acute injury swelling, tissues were fixed at two different time points after needle retraction, 10 and 25 min. Bilateral insertion experiments were conducted on each rat. The second retraction was 15 min after the first one. Then, the rat was immediately euthanized by perfusion fixation using 10% formalin which took ∼10 min.

#### Infusion pressure measurement

In-line infusion pressure provided a separate measure of needle-tissue interaction. In previous studies, spikes in infusion pressures have indicated the occurrence of tissue coring within the needle tip [3], [14], [24]. In-line pressure measurements were performed for all needle insertion experiments to monitor pressure changes during insertion and CED at 2 µL/min.

Pressures were measured using a custom-designed fiber optic pressure transducer (model FOP-MIV-NS663, FISO Technologies, Quebec, CA) that was connected in-line to the infusion system between the syringe and the needle. Pressure was monitored at 1 Hz during the insertion, infusion, and retraction process. Prior to each brain infusion, a reference pressure was recorded outside the brain with the infusion pump running at the test infusion rate and with the needle at the same stereotaxic level as within the rat brain during CED. This value was a measure of the pressure drop within the infusion line and was subtracted from the *in vivo* pressure measured during infusion in brain to determine pressure at the needle tip.

#### Hole measurements along the needle track

Fixed brains were sectioned into 100 µm slices in the horizontal plane on a cryostat (∼−20°C). Approximately 45 slices were obtained along each needle track. Slices were put in 1× PBS at room temperature for 30 min and then mounted on microscope slides for imaging. Needle holes on each slice were imaged on a microscope (model IX-71, Olympus America Inc., Center Valley, PA) and a digital camera (model SPOT RT3, Diagnostic Instruments, Inc., Sterling Heights, MI) using bright light. Color RGB images of the hole were used to measure the area and the perimeter using a MATLAB script. Images were converted to gray scale and the hole was detected by considering only pixels with intensity higher than a threshold value determined from surrounding tissue in each slice using Otsu's method [36].

Since the holes were not necessarily circular, the measured areas were used to calculate the equivalent diameter, which was defined as the diameter of a circle with the same hole area. The aspect ratio, defined as the ratio between the equivalent perimeter (calculated using the equivalent diameter) and the measured perimeter, was also used to evaluate the shape of the hole. The aspect ratio is 1 for a circular hole, and the aspect ratio goes to zero as the shape becomes more irregular. In some slices, especially those close to the brain surface, the hole was difficult to measure because of excessive bleeding produced during dura mater removal or because the hole was closed. These holes were not included in the data analysis.

### Additional histological assessment

In an additional test group (*n* = 3 rats), needles were inserted at the three insertion speeds and 4 µl of albumin in solution with 1×PBS (20 mg/ml) was infused at 2 µL/min. Evans blue was not infused to allow H&E staining. Fixed brain tissue was sliced into 50 µm thick slices in horizontal planes and standard procedures for H&E staining were followed [23].

### Pre-stress estimation

Hole diameters were input into a tissue mechanics model to estimate pre-stresses along the length of the needle track. The model assumed that during insertion, tissue was radially displaced and conformed around the inserted needle. This is based our assumption that frictional drag of tissue in the z-direction is small along the main length of the needle and that shear stresses become negligible as the tissue equilibrates around the needle before the start of CED. Also, a plane strain condition was assumed because of the relatively large length of the needle with respect to the diameter. In this way, changes in hole diameters can be related to corresponding radially directed tissue stresses at the needle surface. Axial symmetry and a semi-infinite medium were considered because of the relatively small diameter of the needle compared with the surrounding tissue. Tissue stress was determined at equilibrium after needle insertion; therefore viscoelastic effects were not considered. Pre-stress was calculated at the most immediate tissue fixation time point (10 min) when swelling was minimized. Since swelling and pre-existing residual stresses can change the hole size, the approach to estimating pre-stress considers the net effect of pre-existent stresses and stress generated during needle insertion.

**Figure 3:**
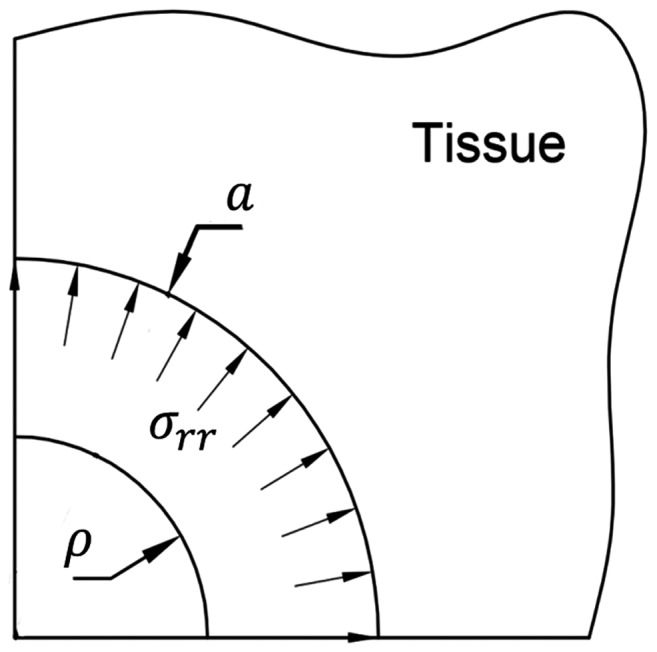
Schematic showing the relation between holes left in tissue after needle retraction and pre-stresses at needle-tissue interface when the needle is in tissue. The hole of diameter ρ can be expanded to the diameter of the needle (*a*) which introduces compressive pre-stress (σ*_rr_*).

Pre-stress was calculated as the compression load on the surface of the hole necessary to re-expand the equivalent hole diameter (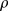) to the diameter of the inserted needle (*a*) (**Figure 3**) assuming the solid phase of tissue behaves as a neo-Hookean material [25], [37]. By assuming incompressibility of the tissue [38], the Cauchy stress tensor was calculated using [39]

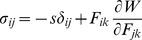
(1)where *s* is a material constant, 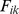 is the gradient deformation tensor, and 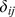 is the Kronecker delta tensor. The simplified energy function was
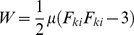
(2)where *μ* is the shear modulus.

By considering plane strain conditions, stretch ratios were defined as
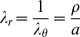
(3)


The constant *s* was calculated far away from the needle where stress and displacements go to zero, and the stretch ratio was equal to unity. From Equation 1, *s* was equal to *μ*

After calculating the derivative of *W*, the stress tensor (Equation 1) becomes
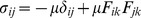
(4)


The pre-stress is the normal stress in the radial direction
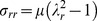
(5)


Pre-stress was calculated using equilibrium shear moduli values of 485 Pa for the cortex and 238 Pa for the corpus callosum using values reported by Elkin et al. [40]. In this case, the external capsule was assumed to have similar modulus to the corpus callosum which is a predominately white matter region. This white matter shear modulus was assigned at two points, 2.8 and 2.9 mm depths, corresponding to the ∼0.2 mm thick layer of external capsule according to a rat brain atlas [41]. All other points were calculated with the modulus of cortex gray matter.

### Backflow experiments

A separate set of experiments was conducted to measure backflow at each insertion speed (0.2, 2, and 10 mm/s). 4 µL of EBA was injected at three flow rates (0.5, 1, and 2 µL/min). Five experiments were performed for each experimental condition for a total of 45 experiments (23 rats with bilateral injections). Following CED experiments, rats were immediately euthanized and freshly excised brains were sectioned into 100 µm slices on the cryostat in the coronal plane. Slices within infused brain regions were mounted on microscope slides and placed on a white surface. White light was projected on each slice, then slices were imaged using a CMOS camera and saved as a RGB file for analysis.

Backflow was quantified by using two different measures: (1) the percentage of tracer infusate outside of the targeted CPu region and (2) the distance from the needle tip to the point of maximum tracer penetration back along the needle track.

#### Backflow as percentage of tracer infusate outside the target

Previous CED studies have calculated the infused volume using a threshold intensity value and assuming constant concentration in regions with intensity above this threshold. This approach can introduce some error since the concentration of infusate is not necessarily constant throughout infused brain tissues. To avoid this, slice images were converted into concentration maps of Evans blue, which was the visible component of the tracer. The distributed tracer regions were used to calculate the mass of tracer outside the target CPu.

To obtain a gray scale image of each slice and calculate a pixel intensity, the red value (*R*) at every pixel was normalized by the total pixel intensity [10], 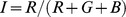 where *G* is the green component and *B* is the blue component of the white light. Images were analyzed using a Matlab subroutine, and pixel intensity was converted to dye concentration using a modified Beer's law [42]

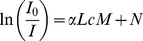
(6)where *L* is the path length of light inside the specimen, 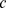 is the Evans blue concentration, 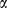 is the absorption or extinction coefficient, and *I_o_* is the incident light energy. Beer's law was modified to account for non-transparent tissue such that *M* is an introduced factor that accounts for the increase in path length of photons that are detected but take a non-straight path, and *N* is a factor which accounts for the scattering of light. The modified law can then be written as [42]

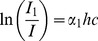
(7)where 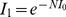, 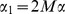, and 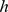 is the thickness of the slice, 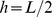 because the light was assumed to cross the specimen then reflect back to the camera. The constant 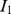 is thus the intensity of the background and was obtained by applying Equation (7) to a reference tissue region without any tracer.

**Figure 4:**
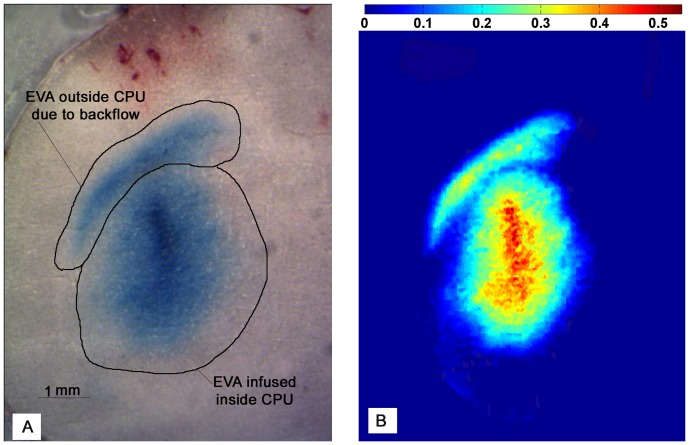
Brain tissue slice showing (A) EBA distribution and (B) corresponding tissue concentration map. The concentration map of Evans blue is in units of mg/mm^3^. The needle was inserted at a speed of 0.2 mm/s and CED was at a flow rate of 1 µL/min.

Next, the mass of Evans blue in a given tissue region was determined in terms of image intensity using mass conservation
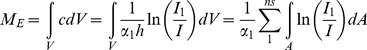
(8)where 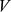, 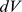, *A*, 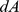, and 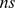 are volume, differential of volume, area, differential of area, and number of slices, respectively. This relation assumes no capillary clearance or metabolism of EBA over the course of the experiment. To obtain the constant 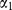, the total mass of Evans blue introduced into the brain by CED (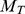 1.25 mg/mm^3^×4 µL = 5 mg) was compared with intensity data from all slices within the infused region. Once the constants 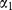 and 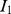 were obtained, Equations 7 and 8 were used to estimate EB concentration and mass accumulated in tissues. An example of a tissue slice image and corresponding Evans blue concentration map within the infused region are shown in **Figure 4**. CPu and white matter regions where easily delineated on each slice as shown in **Figure 4A**. It can be seen that concentration gradients existed inside the CPu and concentrations were not constant (**Figure 4B**).

The backflow percentage (*BF*) was calculated as
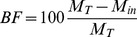
(9)where 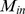 is the mass of Evans blue inside the target CPu. The region with EBA inside the CPu was isolated on each slice using ImageJ software (National Institutes of Health). The mass of Evans blue outside the CPu was not calculated directly from the images because blood was frequently observed outside the CPu, especially in white matter regions.

#### Backflow distances

**Figure 5:**
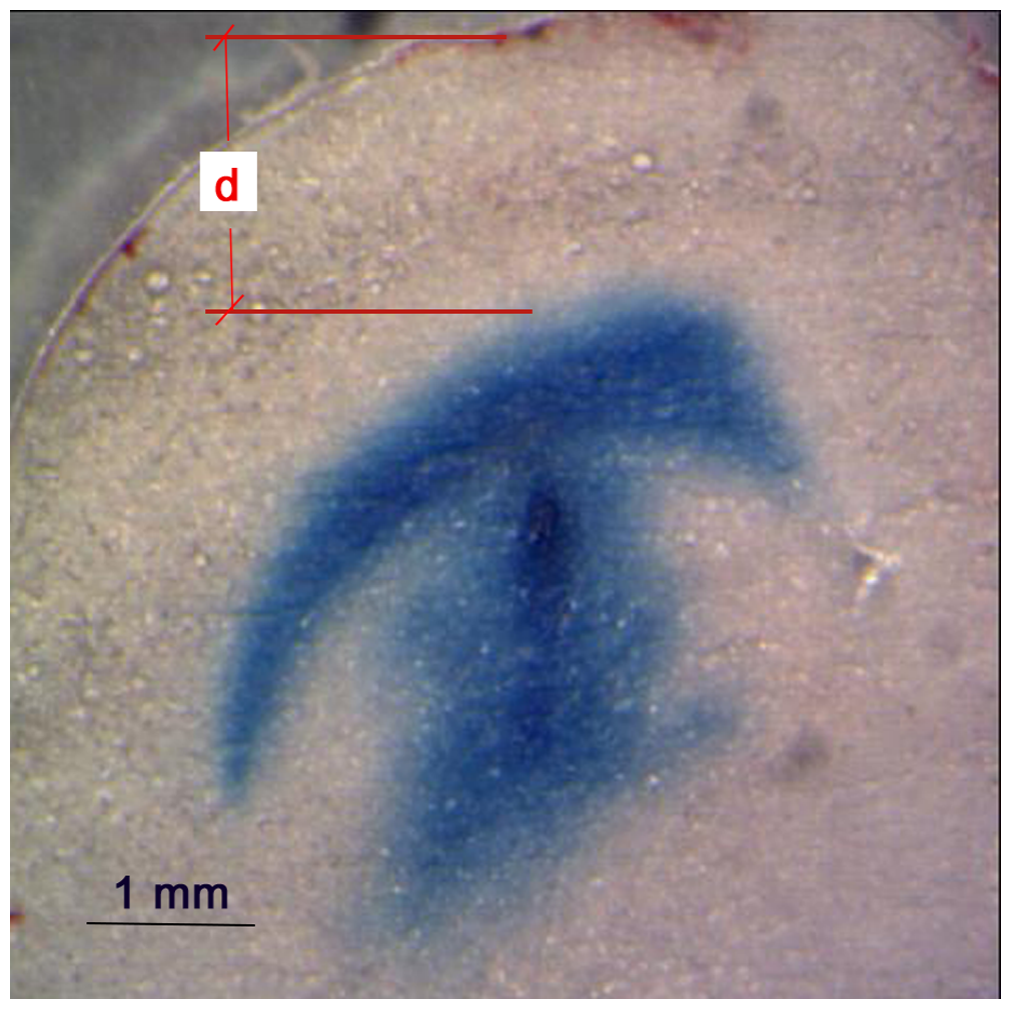
EBA backflow distribution along the needle track. For backflow calculations, distance along the needle track from the brain surface to the top most backflow region (*d*) was measured. Image is of an excised 100 µm brain slice.

Backflow distances from the needle tip to the point of maximum tracer penetration back along the needle track were also determined. For each infusion site, a slice containing the needle track or as close as possible to it was used. Because the position of the needle tip was difficult to identify in the brain slices, backflow distances were calculated as the difference between the total insertion depth (5 mm) and the distance (*d*) from the point of maximum dye penetration along the needle track to the surface of the brain, see **Figure 5**.

### Statistical analysis

Analysis of variance (ANOVA) was used in the analysis of experimental data to test whether or not the means of several groups are all equal. One-way ANOVA was used to examine the influence of one factor on the experimental data and two-way ANOVA was used to evaluate the effect of two or more factors. The results of ANOVA can be considered reliable as long as the data are normally distributed. If normal distribution could not be guaranteed, Kruskal-Wallis test was used.

Average infusion peak pressure and steady state pressure was compared for the three insertion speeds by using one-way ANOVA. The total average hole areas, diameters, and pre-stresses, were compared for the three insertion speeds and the two time points by using the Kruskal-Wallis test because data was not normally distributed. Also, the Kruskal-Wallis test was used to compare averages areas, diameters, and pre-stresses among regions in the brain along the needle track (cortex, external capsule, and CPu). Average hole area for each point along the insertion track was compared for the three insertion speeds and for the two time points by using two-way ANOVA. At some depths along the needle track, the hole was difficult to see in images and was not measurable. Therefore statistical analysis was performed only for those depths where the complete data set which consisted of four repetitions was obtained. Average backflow percentage and backflow distances were compared for the three insertion speeds and the three flow rates by using two-way ANOVA. Data are presented as mean ± 1 standard deviation. All p-values of <0.05 were considered significant.

## Results

### Damage assessment

#### Infusion pressure

**Figure 6:**
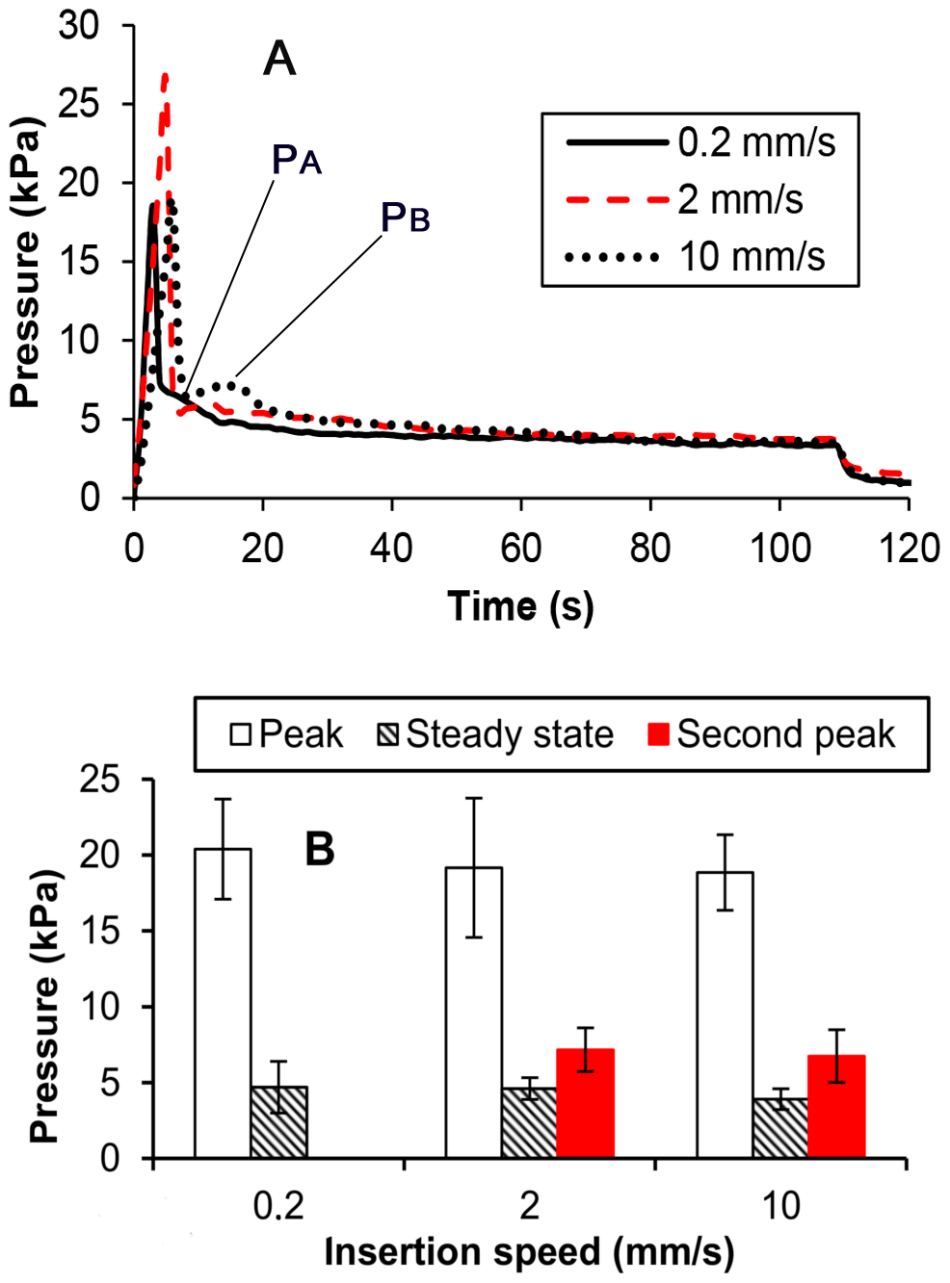
Insertion speed dependence of infusion pressure for CED at 2 µL/min; A) typical pressure profiles during infusion; B) average peak, steady state pressure, and second peak (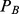) for varying needle insertion speeds. (Bars indicate ±1 standard deviation; *n* = 8).

Small variations in infusion pressure were observed during needle insertion. Once infusion started, pressure increased to a peak value between 14 and 25 kPa and then decreased to an approximately steady-state value between 2.7 and 7.4 kPa (**Figure 6A**). The peak pressure may be indicative of tissue coring or some other initial tissue resistance which the infusate encounters. After infusion was stopped, infusion pressure decreased to a value just above zero and then went to zero after the needle was fully retracted. For infusions performed with needle insertion speeds of 2 and 10 mm/s, a second small peak (*P_B_* in **Figure 6A**) between 0.2 and 2.46 kPa greater than the local minimum value (*P_A_*) was also observed.

**Figure 6B** shows the average values of peak pressure, steady state pressure, and the second peak of pressure (*P_B_*) for the different insertion rates. The difference between average peak and steady state values was not significant with respect to insertion speed (p-value = 0.62 for peak pressure; p-value = 0.29 for steady state pressure). That peak pressure did not depend on insertion speed indicated that tissue coring or tissue resistance due to tissue separation was not insertion rate dependent. The difference in the second peak between insertion speed 2 and 10 mm/s was not significant (p-value = 0.6). Magnitude of the second peak of pressure was given by the difference 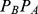. The average of that difference was 0.54 and 0.84 kPa for 2 and 10 mm/s insertion speeds, respectively. The averages of these magnitudes were also not significantly different (p-value = 0.39).

#### Hole measurements

**Figure 7:**
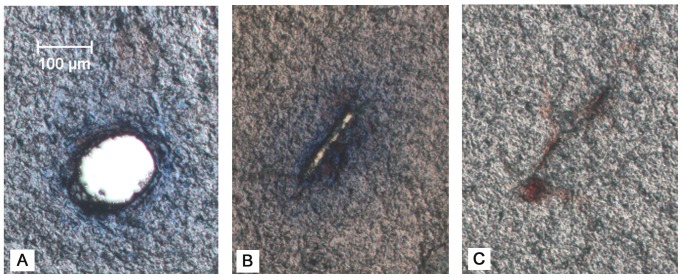
Different types of tissue damage left after needle retraction. A) approximately circular hole surrounded by infused EBA; B) hole as a slender opening/crack; and C) red blood cell accumulation where it was difficult to measure the hole opening. All images are for fixed, 100 µm thick brain tissue slices.

In the majority of tissue slices, the hole left by the needle appeared as empty space (**Figure 7A**), but in other slices (∼5% of slices), especially those close to the surface on the cortex, the hole appeared as a narrow crack surrounded by EBA (**Figure 7B**). In other cases (∼20% of slices), only a region with accumulation of red blood cells was observed (**Figure 7C**) where it was difficult to distinguish the actual track of the needle. These corresponded mainly to locations close to the surface of the brain at insertion depths shallower than 1.4 mm. Data from slices like those shown in Figure 7B and 7C were not considered in any data analysis. Therefore, ∼36 hole measurements per needle track were considered.

**Figure 8:**
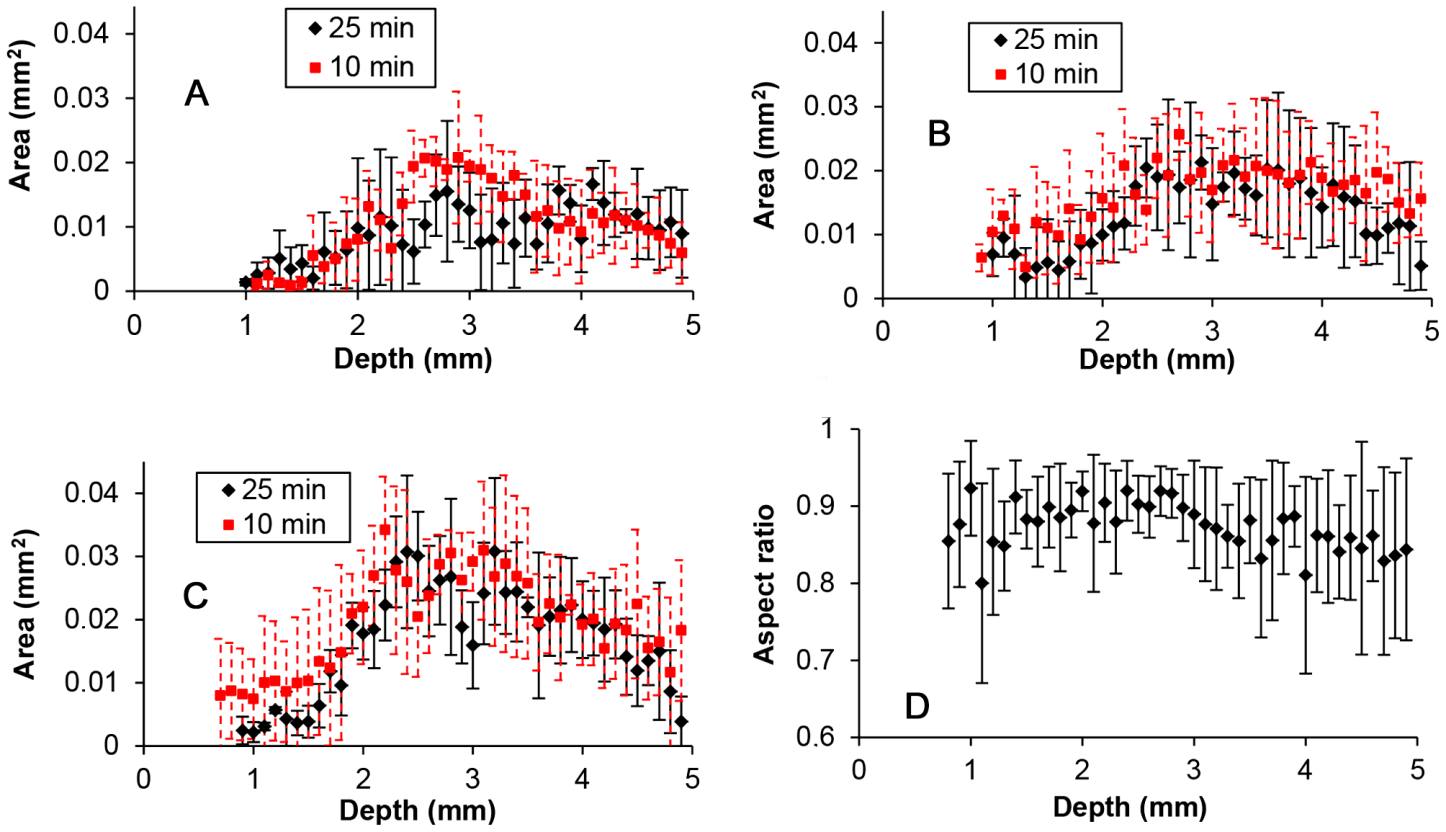
Tissue penetration damage along the length of the needle track. Areas of holes left after needle retraction are plotted as a function of insertion depth for varying fixation time points (10 and 25 minutes) and insertion speeds: A) 0.2 mm/s; B) 2.0 mm/s; and C) 10 mm/s. D) Aspect ratio of holes for an insertion speed of 0.2 mm/s and fixation after 10 min. Each data point corresponds to the average of 4 hole measurements. Bars represent ±1 standard deviation.

Measured hole areas were averaged at each spatial location along the needle track for each insertion speed and fixation time point, **Figure 8**. Hole area varied spatially with insertion depth. Track holes increased in size with penetration in the cortex. Between 2 and 3.5 mm depth, hole size reached a maximum, and decreased again for higher depths. The region of maximum hole area corresponded to the white matter region (external capsule). For two experiments performed at 10 mm/s insertion speed, the area of the hole within white matter regions was practically the same as the cross area of the needle (0.0434 mm^2^). For the rest of the measured slices, the area of the hole was always considerably smaller than the area of the needle. **Figure 8D** shows the aspect ratios of the holes along the needle track for the 0.2 mm/s insertion speed. Holes were more circular (higher average value and lower standard deviation) between depths of 2 and 3 mm. Holes were found to be less circular at the CPu target site and near the surface of the brain. The aspect ratio profiles for other insertion speeds were found to be similar and are not shown.

**Figure 9:**
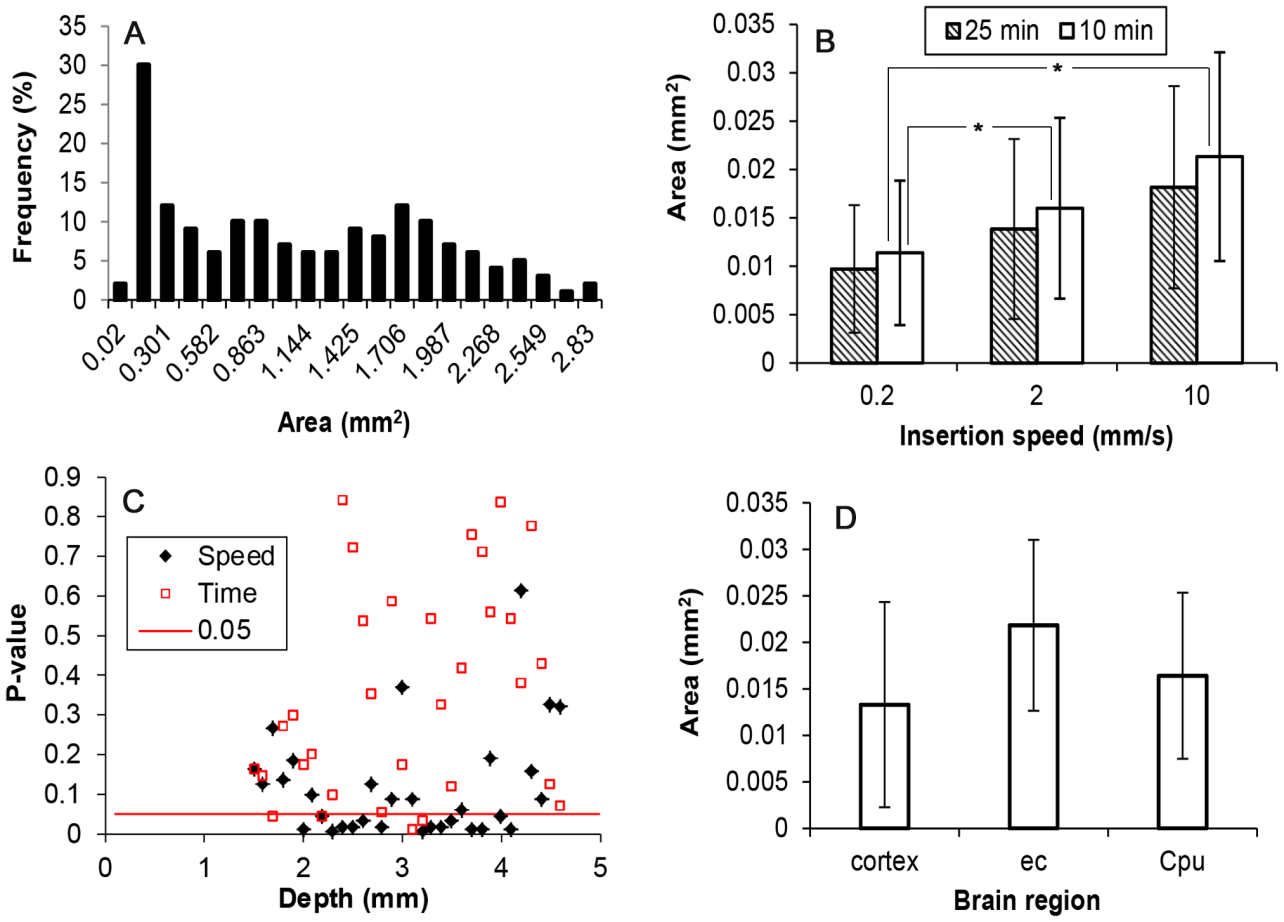
Variation of hole measurements. A) histogram showing non-Gaussian distribution of the hole areas for 10 mm/s needle insertion speed and fixation after 10 min (combined data from 4 needle tracks, 161 total hole measurements); B) average hole areas for the three insertion speeds and the two fixation time points evaluated for depths greater than 1.4 mm. Each bar corresponds to the average of *n* = 144 hole measurements. Significant difference with 10 mm/s is noted by *; C) analysis of variance to evaluate the influence of insertion speeds (0.2, 2, and 10 mm/s) and the influence of fixation time point (10 and 25 min). p-value is plotted for varying insertion depths; D) average area of holes for main tissue regions along the needle track. Each average value was significantly different from each other. Each bar corresponds to the average of *n* = 336, *n* = 48, and *n* = 504 measurements for cortex, external capsule (ec) and caudate putamen (CPu) regions, respectively. For cortex, points between 1.4 and 2.7 mm depth were considered. For external capsule, points at depth 2.8 mm and 2.9 mm were considered. For caudate putamen, points at depth higher than 2.9 mm were considered. Bars represent ±1 standard deviation.

Areas of all the holes along the needle track for each insertion speed and time point were averaged and compared by using the Kruskal-Wallis test (**Figure 9A**). As shown in **Figure 9B**, the average area increased for increasing insertion speed, see **Table 1**. Also, average hole area was larger for the earlier fixation, 10 min time point. The total average area for the 10 min fixation time point (0.01624±0.010 mm^2^) and for the 25 min time point (0.0139±0.009 mm^2^) was also significantly different. Changes in hole area were more sensitive to changes in insertion speed than to fixation time point; the average area for 10 mm/s was 1.87-fold and 1.32-fold the average area for 0.2 mm/s and for 2 mm/s respectively, while the average area for 10 min before fixation was up to 1.17-fold the average area for 25 min after fixation.

**Table 1:** Needle insertion and CED results for the evaluated needle insertion speeds.

Insertion speed	0.2 mm/s	2 mm/s	10 mm/s
**Hole area** * **(mm^2^)**	0.0105±0.007	0.0149±0.009	0.0197±0.010
**Diameter** * **(mm)**	0.107±0.042	0.137±0.047	0.152±0.046
**Perimeter** * **(mm)**	0.383±0.145	0.483±0.153	0.536±0.150
**Average pre-stress (Pa)**	351.7±92.4	266.7±109.3	240.0±125.1
**Backflow percentage** ** **(%)**	17.07±11.7	27.7±11.6	39.4±10.4
**Backflow distances** ** **(mm)**	2.82±0.17	2.86±0.12	2.80±0.14

Average values ±1 standard deviation are presented.

*average for 10 and 25 min fixation time points.

**average for 0.5, 1.0 and 2.0 µL/min flow rates.

Analysis of variance was performed to compare the area of the holes at each point along the needle track, see **Figure 9C**. With respect to insertion speed, difference was significant for the majority of points between 2 and 4 mm depths. Here, increased insertion speed produced increasing hole areas except within a narrow region at ∼3 mm depth which likely corresponds to the white matter region. With respect to the fixation time point, hole differences were significant for 12.5% of depth locations.

**Figure 9D** shows average hole areas for the three main brain regions along the needle track. Average values for the cortex (0.0133±0.011 mm^2^), external capsule (0.0218±0.009 mm^2^) and CPu (0.016±0.008 mm^2^) were significantly different from each other. The maximum difference in average area was between the external capsule and cortex where average area of the external capsule was 1.64-fold the average area in the cortex. This ratio is smaller than for the ratio between 10 mm/s and 0.2 mm/s insertion speeds. Therefore, tissue damage was more sensitive to insertion speed than brain region over the range of parameters tested.

Average hole diameters and perimeters presented similar trends as the hole areas. These results are summarized in **Table 1**.

#### Additional histology

**Figure 10:**
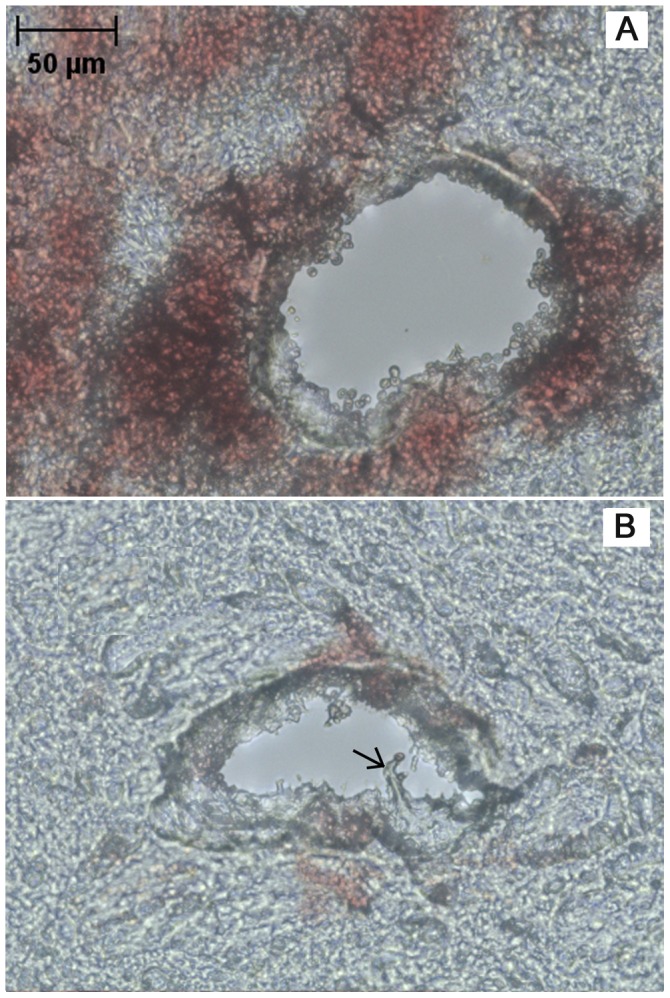
Needle tissue damage at the hole surface. A) cell separation and bleeding in the external capsule for insertion at 10 mm/s, and B) torn fibrous tissue (arrow) in the CPu for insertion at 0.2 mm/s. Both images are for 50 um thick fixed brain tissue slices with no staining.

**Figure 11:**
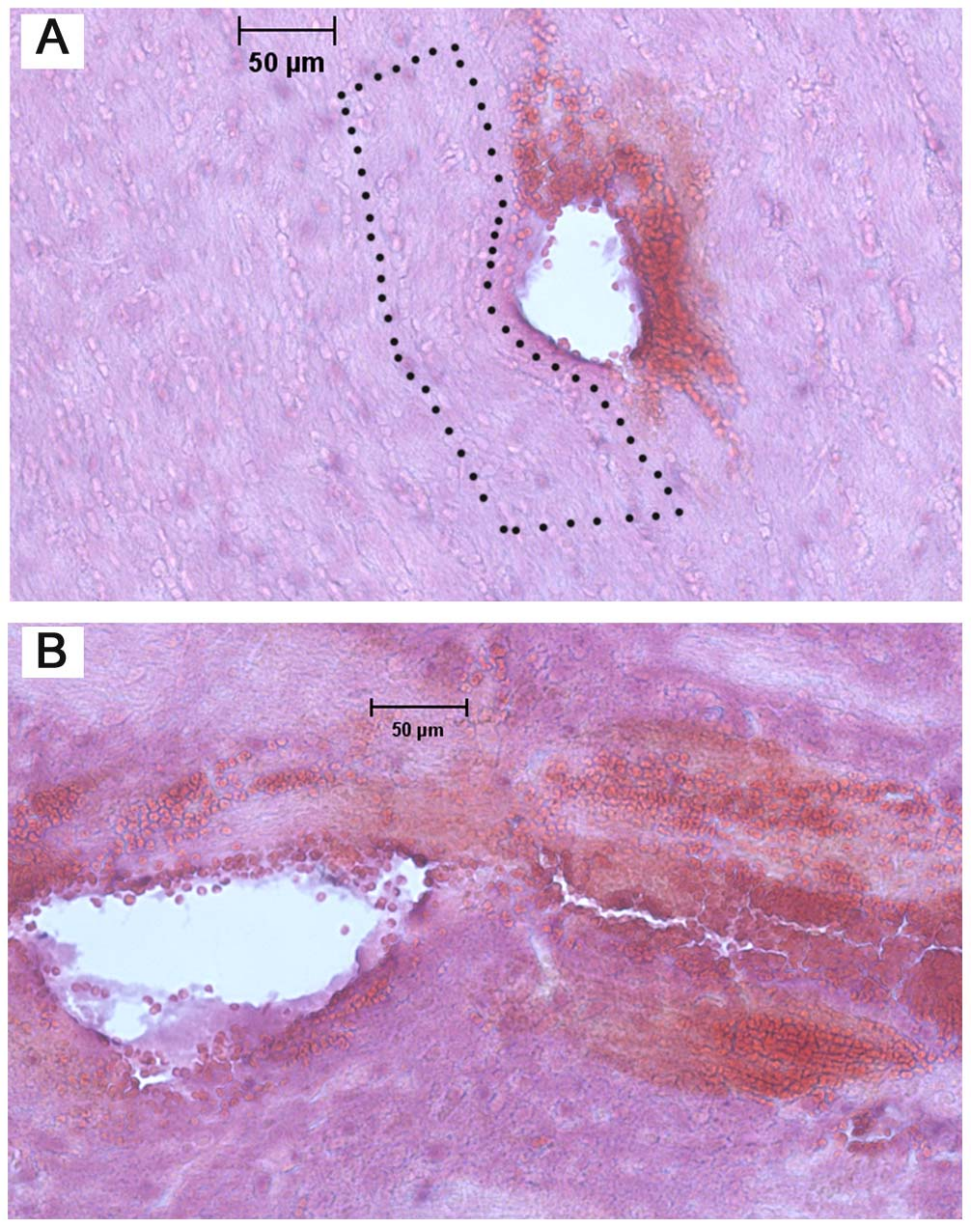
Needle tissue damage observed with H&E staining. A) external capsule region showing deformation and compaction of surrounding tissue. In the enclosed area (dotted lines), layers of cells conform around the hole (needle insertion at 0.2 mm/s); B) CPu region showing extensive bleeding and tissue fracture at some distance away from of the hole (insertion at 10 mm/s).

Several rupture mechanisms were observed in brain tissue slices. Tissue fracture was defined as a crack or narrow opening with bleeding in interior regions of the tissue slice. The presence of blood indicated that fracturing occurred *in vivo* during needle insertion or retraction and not during tissue processing, e.g. slicing and mounting. **Figure 10A** shows tissue fracture and tissue stained with blood close to the needle hole. Generally, this injury was observed mainly in white matter regions of the external capsule and in the CPu. More intensive bleeding and tissue fracturing was also often observed at higher speeds, 2 and 10 mm/s (**Figure 11B**). Tissue fractures between 20 and 150 µm in length were found. This type of damage was observed in approximately 35% of the needle insertion experiments conducted at 2 or 10 mm/s. At the slower insertion speed, bleeding was only observed close to the hole, and tissue fracturing was not observed.

Individual cell separation from the bulk of tissue was also frequently observed at the surface of holes (**Figure 10A**). Narrow strips of tissue were also separated from the tissue surface (arrow in **Figure 10B**). Layers of cells or fibers were occasionally observed to be curved at the hole boundary, especially at slower insertion speeds of 0.2 mm/s (**Figure 11A**). This provided some evidence of radial tissue compaction.

### Pre-stress

**Figure 12:**
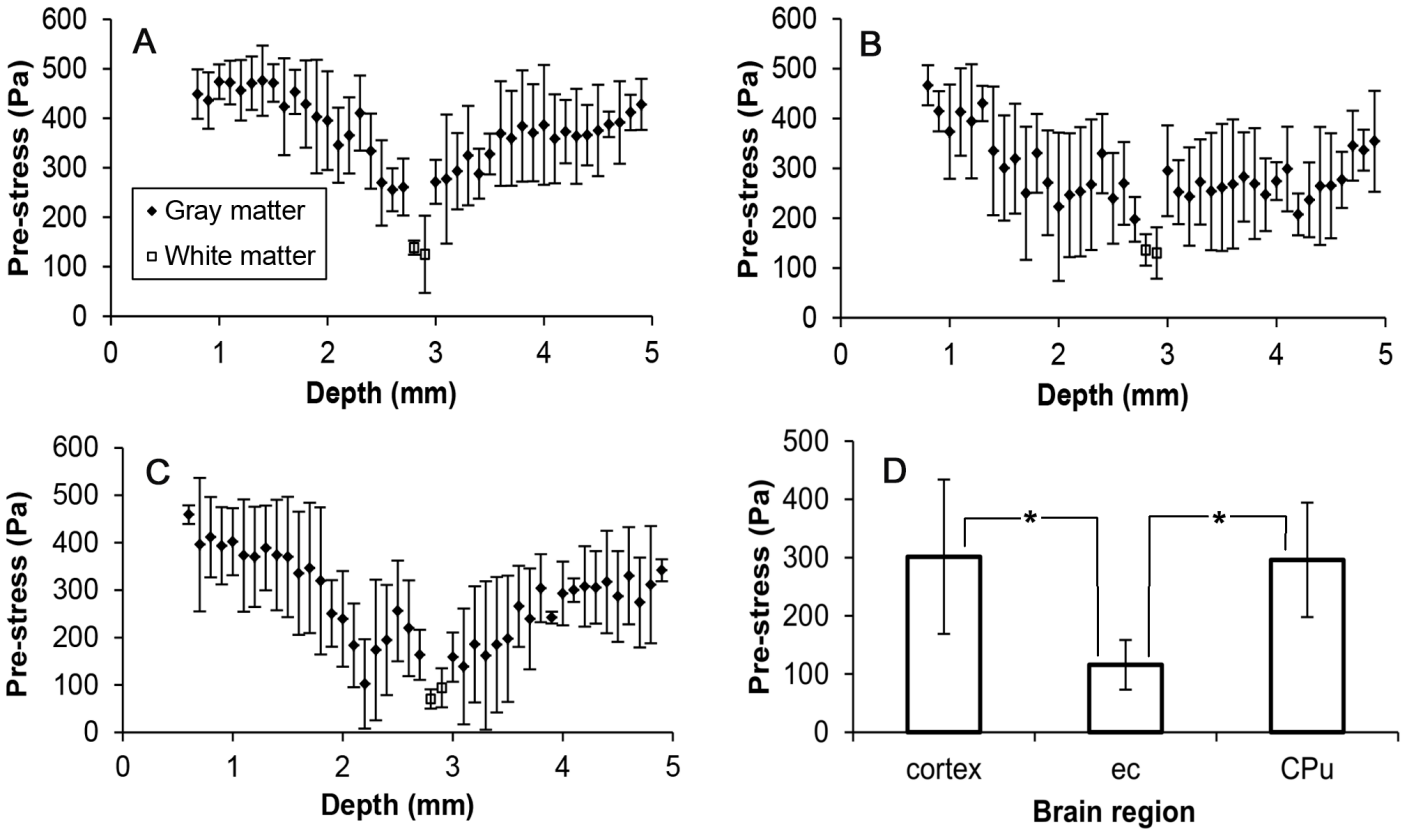
Pre-stress along the needle track for the needle inserted at: A) 0.2 mm/s, B) 2 mm/s, and C) 10 mm/s. (Bars represent the calculated standard deviation, *n* = 4.) D) Average pre-stress values for cortex, external capsule, and CPu regions. Significant difference is noted by *, p-value<0.05. Each bar corresponds to the average of *n* = 144 pre-stress values.

Pre-stress was calculated from equivalent hole diameters at the earliest fixation time point for each of the three insertion speeds, see **Figure 12**. Obtained pre-stress values were between 0 and 485 Pa. Pre-stress in white matter was approximately zero at five locations corresponding to locations where the area of the measured holes were practically the same as the needle. Average pre-stress values along the entire needle track are presented in **Table 1**. The average pre-stress for 0.2 mm/s was found to be significantly higher than pre-stress averages at the other two insertion speeds. There was no significant difference between pre-stress averages at 2 and 10 mm/s insertions (p-value = 0.16).

Pre-stress was not constant along the needle track. Maximum values were found in the cortex close to the surface, minimum values were found in the external capsule white matter region, and pre-stress increased with greater depths beyond the external capsule. Average pre-stress values for cortex, external capsule, and CPu regions are shown in **Figure 12D**. The average pre-stress value for the external capsule was significantly different from the other two tissue regions. There was no significant difference between average pre-stress in the cortex and CPu (p-value = 0.5).

### CED backflow distributions

**Figure 13:**
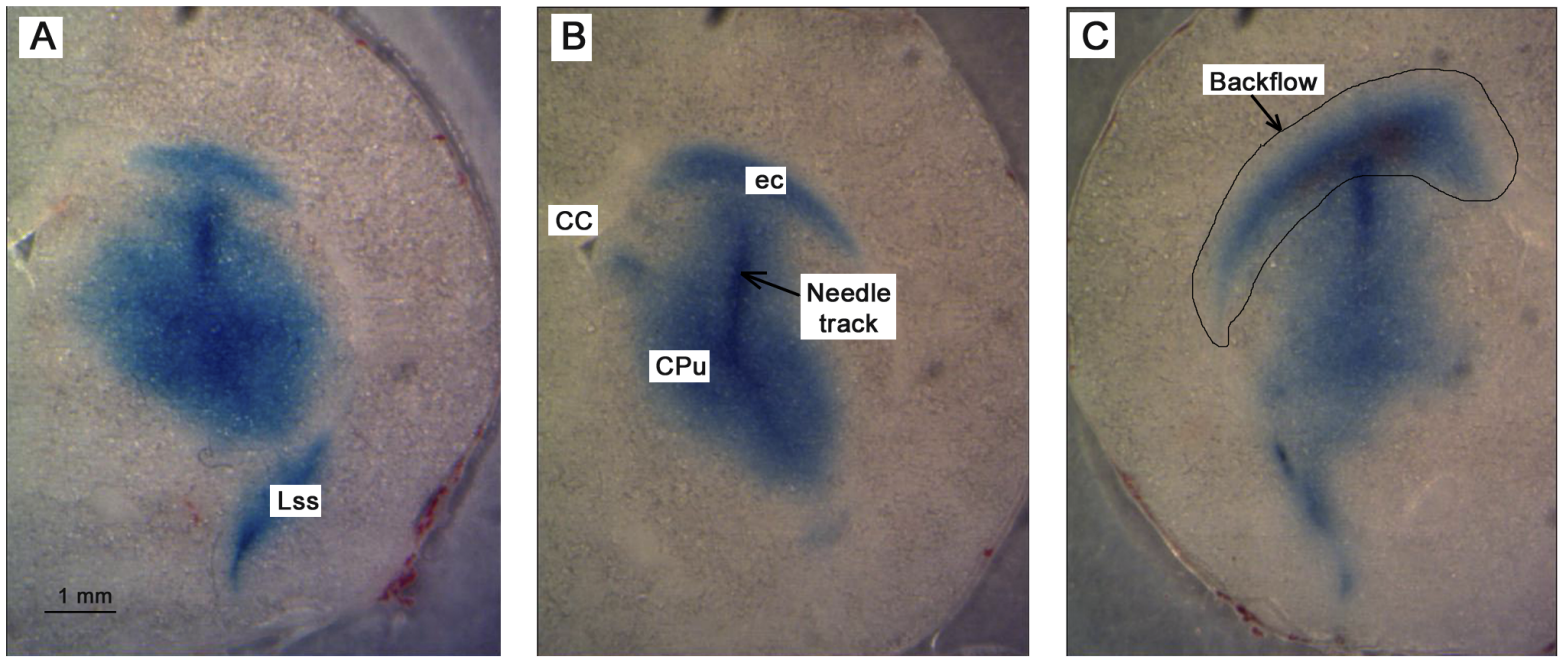
CED backflow in the rat brain. Images show distributions along the needle track after infusion of 4 µL of EBA at 0.5 µL/min. Infusion needles were inserted at varying speeds: A) 0.2 mm/s, B) 2 mm/s, and C) 10 mm/s. Rat brain slices are 100 µm thick slices and unfixed (ec: external capsule, CPu: caudate putamen, Lss: lateral stripe of the striatum, cc: corpus callosum).

CED distributions in brain for varying needle insertion speeds are shown in **Figure 13**. Tracer distributions in tissue were found to be relatively uniform over regions of convective transport with evidence of diffusion at the peripheral front. Backflow was observed as leakage of infusate into white matter regions of the external capsule and corpus callosum (cc) outside of the targeted CPu. Infusate was not observed to flow back into the cortex. In general, backflow increased with increasing insertion speed. In these experiments, evidence of bleeding was frequently found within the external capsule, especially for insertions at 10 mm/s.

Leakage of infusate into the lateral stripe of the striatum (Lss), as shown at the bottom of **Figure 13A, and 13C**, was also frequently found (∼65% of cases) independent of flow rate or insertion speed. Leakage to the lateral ventricles was observed in two cases performed at an insertion speed of 10 mm/s. No evidence of tracer transport directly between Lss and external capsule regions was observed in any of the slices. For that reason, tracer distributions in the Lss were not considered in backflow calculations.

**Figure 14:**
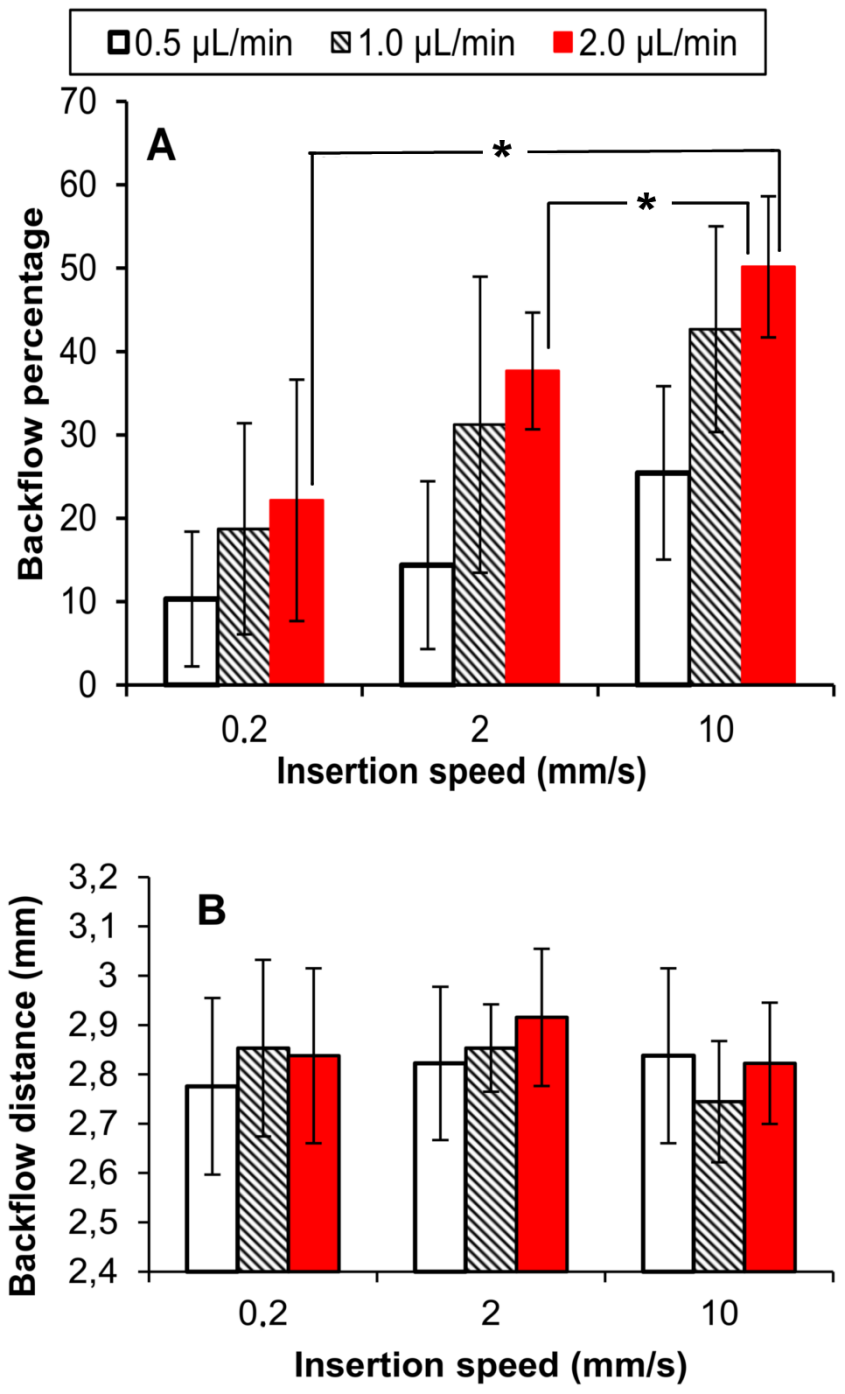
CED backflow following infusion of 4 µL of EBA. A) percentage of the EBA infusate outside the target CPu. Significant difference is noted by *, p-value<0.05 only for 10 mm/s insertion speed. For other flow rates, only the difference between 0.2 mm/s and 10 mm/s was significant. B) Backflow distances. Bars indicate ±1 standard deviation; *n* = 5 for each test group.

Average backflow percentages for varying insertion speed and infusion rate are shown in **Figure 14A**. Significant influence of the flow rate and the insertion speed was found. Average backflow percentages for the three insertion speeds were 16.7, 30.8, and 36.6% for flow rates of 0.5, 1, and 2 µL/min, respectively. Average backflow at 0.5 µL/min was significantly different from the average backflow at the other two infusion rates.

Average backflow percentage was also found to increase for increasing insertion speed, see **Table 1**. The average percentage for the three flow rates were all significantly different at the three insertion speeds. Comparing backflow percentages obtained at 10 mm/s with respect to 0.2 mm/s insertion speeds, backflow increased by 2.46, 2.27, and 2.26-fold at 0.5, 1, and 2 µL/min flow rates, respectively. Since backflow was diverted away from the needle track into white matter regions, backflow distances along the needle track were relatively constant over the insertion speeds and flow rates tested (**Figure 14B**). No significant differences between flow rates or insertion speeds for backflow distance was found (p-value = 0.52 for insertion speeds, and p-value = 0.65 for flow rates).

## Discussion

In this study, brain tissue damage and backflow during CED was evaluated for varying needle insertion speeds. Histological assessment and hole measurements were used to characterize local tissue damage along needle insertion tracks. This unique approach to characterizing tissue disruption was also used to estimate tissue stresses introduced by needle insertion (pre-stress). To the best of our knowledge, this is the first study to estimate pre-stress around an implant in brain tissue, and this information can be used to improve needle implantation surgical procedures or to improve prediction of backflow.

Tissue damage, evaluated as the size of the hole left by the needle after retraction, bleeding, and tissue fracturing, was found to increase for increasing insertion speeds and was higher within white matter regions. A statistically significant difference in hole areas with respect to insertion speed was found. While there are no previous needle insertion speed studies with which to directly compare, previous electrode insertion studies have noted greater brain surface dimpling and insertion forces with increasing insertion speed [43]–[45]. These higher deformation and force measures may indicate greater brain tissue damage which is in agreement with the present study. There are also studies which have found that fast insertion of sharp tip electrodes produced less blood vessel rupture and bleeding [28], [29]. These differences in rate dependent damage may be due to differences in tip geometry (diameter and tip) or tissue region, since these electrode studies focus mainly on the cortex [28], [29]. In the present study, hole measurements were small in the cortex, and no substantial bleeding was observed in the cortex except when it was produced during dura mater removal. Any hemorrhage was observed primarily in white matter regions of the external capsule and the CPu.

### Hole measurements

The hole left in tissue by the needle provided a visual way to reconstruct tissue damage along the needle track. Holes area were relatively small in the cortex, maximum within the white matter region and decreased again in the CPu. The decrease in hole area in the CPu indicated that damage did not accumulate with depth, and that there was negligible tissue accumulation around the needle tip. Hole measurements were more circular in shape within inner cortex and white matter regions and more irregular close to the brain surface and within the CPu. The irregular shape of the hole may have resulted in non-uniform pre-stresses which may have increased the likelihood of backflow in less compacted regions at the needle periphery, i.e., regions of low pre-stress. The variation of the hole area along the needle track is likely because of differences in tissue composition, mechanical properties (modulus, friction, and rupture strength), as well as pre-existing residual stresses in the brain. In a previous study by Xu et al. [26], white matter corpus collosum was reported to be under tensile residual stress that was up to 1.2 kPa. The larger diameter holes observed in the white matter regions of this study may be due to in part to this tensile residual stress.

Local tissue damage was also assessed. More intensive bleeding around the needle was found in white matter regions which is in agreement with hemorrhage patterns seen by White et al. in their CED studies into the rat CPu [23]. This indicates that vasogenic edema is likely. Also, widespread damage to both ECM and cells is possible. A corresponding observation was tissue fracturing observed primarily within white matter regions and the CPu. This is in partial agreement with the study by White et al. [23] where they also reported tissue fracturing but only close to the needle tip (CPu) but not within white matter regions. Greater injury in white matter may be produced by higher rupture stresses as suggested by previous probe insertion force measurements where significant increases in insertion force where found along white matter tracks [43], [44]. Larger deformations associated with greater tissue strength may be the cause of more damage in these regions.

With respect to the two fixation time points evaluated, significant difference in the hole size was found. Swelling, inflammation or other injury processes may be increasing tissue volume after initial injury resulting in the measured decreases in hole areas. These results are in agreement with previous studies where brain tissue swelling and increases in intracranial pressure were detected at early time points after injury [30]–[32]. Therefore, hole measurements include the combined effects of tissue swelling, tissue tearing due to needle insertion, and residual stresses. Reported pre-stress values therefore account for swelling at these specific fixation time points and can be applied to tissue-needle interactions at these time points. Volumetric increases in tissue with swelling will increase pre-stresses with time. If these changes are large enough, they may reduce backflow. However, previous CED studies have not found additional wait time before infusion to be an important influence in preventing backflow [4]. It should be noted that hole damage was found to be less sensitive to changes in fixation time point than to changes in insertion speed.

### Tissue coring

If tissue coring occurs during needle insertion, pressure inside the needle increases at the beginning of the infusion in order to expel cored tissue and clear the needle. Infusion pressures were used to determine if higher insertion speeds produce greater coring as another cause of greater backflow; however, pressure measurements showed no significant difference in the peak expulsion pressures. This indicated that tissue coring was not the main cause of insertion rate dependent changes in backflow. The peak and steady state infusion pressures were greater than or similar to those reported in previous studies [3], [14], [24]. Increased pressures may be due to the use of smaller cannulas which require greater pressure build up than larger cannulas to expel the same volume of tissue. Interestingly, incidence of backflow increased with the appearance of a second small peak in pressure observed at 2 and 10 mm/s insertion speeds. This small peak may be due to dynamic fluid-tissue interactions along the wall or with any cored tissue. Occurrence of a second peak may be a better indicator of backflow than having a high peak pressure value. Decreases in pressure over time may be due to viscoelastic properties of tissue. Slow separation of tissue from the needle (increasing surface area) may also explain decreasing infusion pressure.

### Pre-stress

Volumetric tissue changes due to cellular and tissue disruption, tissue swelling and residual stresses ultimately influences internal tissue stresses surrounding the implanted needle. Improved understanding of theses stresses is needed for improved understanding of tissue-infusate interactions that affect backflow. This study provided measure of changes in pre-stress with changes in insertion speed and brain region. Calculated pre-stress was found to be significantly smaller for high insertion speeds because of the greater tissue damage produced at these speeds. Also, pre-stress was determined to be non-uniform along the needle track. It was smallest within the white matter region of the external capsule and it was predicted to be always present and compressive within the cortex and targeted CPu. Thus, the idealized model predicted no gaps between the needle and the tissue within the cortex and CPu. Predicted pre-stress values were low (<500 Pa) in part due to the low shear modulus values of brain tissue and the large extent of tissue disruption introduced by the needle. These low pre-stress values explain the sensitivity of CED to backflow since there is less of a ‘seal’ at the tissue-needle interface. Consistent with this, calculated pre-stresses were smaller than measured infusion pressures. Applied to CED outcomes, regional variation in pre-stress also supports the observed backflow patterns where infusate moved back up the needle track in the CPu and diverted mainly into adjacent white matter where less of a ‘seal’ exists. Therefore, pre-stress in the CPu region is the most relevant value when predicting backflow because this is the region where infusion and backflow is initiated.

Reported pre-stress was dependent on the mechanics model chosen and the mechanical properties of brain tissue used. These factors affect the magnitude of the reported pre-stress value. There exist some techniques to directly estimate stress at the tissue-needle interface using probes or microballoons as reported in previous studies [46], [47]. However those devices are too large to be used in the rat brain. Moreover, with these techniques only one average value for stress is usually obtained, and information about regional variation along the needle track is missing. Therefore, the combined experiment and modeling approach used in this study establishes a useful way of estimating pre-stresses variation surrounding needles and other brain implants.

### Sources of error

Friction and adhesion which were not considered in this study are potentially important parameters for understanding mechanics between the needle and tissue during needle insertion. Further studies measuring insertion forces are needed to estimate these effects.

While fixation was introduced to stop tissue swelling, fixation may also produce volumetric tissue contraction [48] which may be a source of hole measurement and pre-stress error. Hole contraction due to the combined effect of fixation and swelling will result in pre-stress overestimation. We estimated the net effect of these sources of error by incorporating volumetric tissue changes of 8% measured in previous fixation studies [48] and our own swelling analysis assuming uniform contraction in the radial direction. The effect of tissue swelling on hole measures was estimated by linear extrapolation of average hole area at 10 and 25 min time points to a zero time point. This adjusted hole area was resulted in a net decrease in pre-stress of approximately 5% which is small. Moreover, because our tissue analysis is a post-mortem technique, hole measures may be sensitive to tissue preparation, e.g. slicing and mounting. However, it is expected that these errors were uniformly spread along the needle track.

### CED Backflow

In all CED experiments, backflow was observed. It was smallest for low flow rate and low insertion speed and significantly increased with higher flow rate and insertion speeds. To induce backflow, tracer infusate likely had to overcome pre-stresses in the CPu to separate tissue and form gap at the needle interface. Once backflow reached the white matter of the external capsule or corpus callosum, it was found to accumulate into these regions. The combination of low pre-stresses and the higher hydraulic conductivity of the white matter with respect to gray matter [49] likely contributed to this diversion of infusate into white matter. There did not seem to be sufficient pressure to open up the tissue gap in the cortex region since no tracers were observed to reach the cortex or the surface of the brain during infusion.

With increasing insertion speed, there was less delivery of EBA to the targeted CPu and greater accumulation in the external capsule and corpus callosum. This reflected greater backflow measured as percentage of the total tracer infused. While backflow distances were not significantly different this was to be expected since tracer was diverted from the needle track to the white matter region. As observed in this study, backflow distances were found to be dependent on the needle pathway and the anatomy of white matter intersection. For this needle pathway, backflow percentage was a better measure of the extent of backflow.

With respect to flow rate, backflow increase with increasing flow rate which was in agreement with numerous previous studies [4], [20], [21]. With respect to insertion speed, the results of the present study are opposite to that of our previous hydrogel study where material damage and backflow increased for a slow needle insertion speed [25]. Since similar experimental parameters (test system, insertion rates, needle tip) were used, the different outcome is likely due to differences in the mechanical behavior of the materials. At the slow insertion speed in hydrogel, an accumulation of material was observed to form at the needle tip which created a larger zone of damage. In contrast, in brain tissue, no tissue accumulation was noted at the tip based on the tissue damage patterns. In both materials there was still correspondence between pre-stress and backflow. The main difference between both materials was the different failure behavior that resulted in different pre-stress states. Brain tissue has been found to fail after high plastic deformation with a fibrous like fracture [50]. On the other hand, hydrogel fracture occurs with low plastic deformation [51].

### Clinical Implications

Backflow has been a persistent problem for CED especially at high flow rates required for clinical applications due to a lack of infusate control. In clinical and primate studies, larger cannulas and designs with step changes in diameter have been used [8], [9], [52], [53]. These needles share the same blunt tip used in our studies which show tissue damage along the needle track to be an important factor affecting backflow. We expect similar dependence of local tissue damage on insertion rate and tissue region. Slow insertion rates that minimize damage should improve targeting. Pre-stresses likely increase with cannula diameter, and increases in tissue compression may partially explain success of step designs in reducing backflow. This effect may also reduce backflow using polymer coated cannulas that swell *in situ* [11]. There are many sources for discrepencies between CED studies (cannula design, cannula alignment, fluidic components, and infusion rates, as well as animal model and infusion site). Tissue rupture and failure initiated at the needle tip is highly dependent on tip geometry, and previous studies have shown sharp or rounded needle tips [20] may reduce the extent of damage and backflow along the needle track. Tissue coring also contributes to pressure build up at the needle tip. Designs that reduce these effects, e.g. side port needles or hollow fiber catheters [10], [54], will also reduce backflow. Further studies looking at the effects of needle tip geometry on tissue damage and stress in the vicinity of the needle tip are needed to better understand infusate-tissue interactions that contribute to backflow.

## Conclusions

In this study, the effect of needle insertion speed on local tissue injury and backflow was evaluated *in vivo* along a track into the caudate putamen. Fast insertion speeds were found to produce more injury including tissue bleeding and disruption, lower pre-stresses, and greater backflow. New pre-stresses values provide important mechanics information at the tissue-needle interface needed to study fluid-solid interactions and may be used in the future as a predictor of backflow. In addition, patterns of tissue disruption and pre-stress were reported to vary along the length of needle track with difference found between white and gray matter regions. Thus, insertion speed dependent damage and changes in pre-stress were found to directly contribute to the extent and patterns of backflow measured. Overall, slower insertion rates resulting in less damage and improved targeting. Future studies should focus on cellular level injury along needle tracks as well as the influence of different CED parameters, e.g. needle tip geometry and needle pathways, on damage and backflow.
